# Long-Term Lung Sequelae in Survivors of Severe/Critical COVID-19 Pneumonia: The “Non-Steroid”, “Non-Interventional” Approach

**DOI:** 10.3390/jcm14020347

**Published:** 2025-01-08

**Authors:** Elvira-Markela Antonogiannaki, Ioannis Grigoropoulos, Effrosyni D. Manali, Konstantinos Thomas, Maria Kallieri, Panagiota Alexopoulou, Andriana I. Papaioannou, Spyridon Prountzos, Anastasia Karachaliou, Christina Kontopoulou, Vagia Karageorgou, Stefanos Lampadakis, Myrto Blizou, Ioannis Tomos, Sotiria Grigoropoulou, Dimitra Kavatha, Stelios Loukides, Anastasia Antoniadou, Spyros A. Papiris

**Affiliations:** 1 2nd Pulmonary Department, General University Hospital “Attikon”, Medical School, National and Kapodistrian University of Athens, 12462 Athens, Greece; kantonogiannaki@gmail.com (E.-M.A.); fmanali@otenet.gr (E.D.M.); mkallieri@yahoo.gr (M.K.); vagiakar@hotmail.com (V.K.); steflamp17@gmail.com (S.L.); myrto_bl@hotmail.com (M.B.); papiris@otenet.gr (S.A.P.); 2 4th Department of Internal Medicine, General University Hospital “Attikon”, Medical School, National and Kapodistrian University of Athens, 12462 Athens, Greece; grigoropoulosioannis@gmail.com (I.G.); costas_thomas@yahoo.com (K.T.); panagiota.alexopoulou@gmail.com (P.A.); grigoropoulou.sotiria@gmail.com (S.G.); dimitra.kavatha@gmail.com (D.K.); ananto@med.uoa.gr (A.A.); 3 1st Respiratory Department, Athens Chest Hospital “Sotiria”, Medical School, National and Kapodistrian University of Athens, 12462 Athens, Greece; papaioannouandriana@gmail.com; 4 2nd Department of Radiology, General University Hospital “Attikon”, Medical School, National and Kapodistrian University of Athens, 12462 Athens, Greece; spyttt@gmail.com (S.P.); anastasia1804@hotmail.com (A.K.); chriskont@hotmail.gr (C.K.); 5 5th Respiratory Department, Athens Chest Hospital “Sotiria”, 11527 Athens, Greece; etomos@hotmail.com

**Keywords:** severe/critical COVID-19 pneumonia, interstitial lung disease, pulmonary fibrosis, computerized tomography patterns, survivor treatment, post-COVID-19

## Abstract

**Introduction**: Long-term lung sequelae in severe COVID-19 survivors, as well as their treatment, are poorly described in the current literature. **Objective**: To investigate lung fibrotic sequelae in survivors of severe/critical COVID-19 pneumonia and their fate according to a “non-interventional” approach. **Methods**: Prospective study of the above COVID-19 survivors after hospital discharge from March 2020 to October 2022. Re-evaluation lasted 3–12 months and included chest HRCT, PFTs, dyspnea, and overall health evaluation by modified Medical Research Council (mMRC) and St. George’s Respiratory Questionnaire (SGRQ), respectively. **Results**: In this study, 198 patients (61.1% male) with a median age of 57 years (IQR 49–66). After 3 months, 187 (94.4%) patients were assessed; after 6 months, 82 (41.1%) patients were assessed; and after 12 months, 16 (8%) patients were assessed. At each time point, a significant reduction was observed in the extent of COVID-19-associated opacities (*p* < 0.001 and *p* = 0.002) and of parenchymal bands (*p* = 0.014 and *p* = 0.025). Persisting fibrotic-like changes were observed in 18 (9%) patients (apical findings in 2 patients, fibrotic non-specific interstitial pneumonia-like changes in 14 patients, minimal fibrotic changes in 2 patients). At 3 months, the predicted median FVC% was 93% (80–100%) and the predicted DLCO% was 65% (58–78%) with a statistically significant improvement at 6 months in both (*p* = 0.001). Moreover, 81.1% had mMRC ≤ 1 and the median SGRQ was 11.65 [0–24.3] with a significant reduction at 6 months in both dyspnea (*p* < 0.001) and SGRQ (*p* = 0.027) persisting at 12 months. **Conclusions**: This prospective study, including only survivors of severe/critical COVID-19 pneumonia, documented the significant improvement in all imaging, functional, and clinical parameters by applying the “non-interventional” approach. These data do not indicate any post-COVID-19 severe/critical pneumonia and “epidemic of widespread pulmonary fibrosis”.

## 1. Introduction

The initial cases of severe acute respiratory syndrome (SARS) related to coronavirus-2 (CoV-2) were identified in China in December 2019, and the virus swiftly disseminated globally [1,2]. To date, over 770 million confirmed cases of COVID-19 and more than 7 million deaths have been reported [3].

SARS-CoV-2 belongs to the betacoronavirus family, and the human-to-human transmission through the respiratory system is the primary mechanism of transmission. The viral spike protein of SARS-CoV-2 recognizes the human angiotensin-converting enzyme II (ACE2) receptor as its own [4]. Within the lungs, the virus binds to type II pneumocytes, leading to renin–angiotensin system dysregulation. The host cell’s transmembrane serine protease type 2 (TMPRSS2) further enhances viral uptake by cleaving the ACE2 receptor and activating the SARS-CoV-2 S protein [5]. This increases pulmonary vascular permeability, leading to pulmonary edema. SARS-CoV-2 also targets pulmonary capillary endothelial cells, thereby amplifying the inflammatory response through the influx of monocytes and neutrophils. The combination of both alveolar damage and a pro-inflammatory state contributes to the development of severe acute respiratory syndrome [5]. The ACE2 receptor is expressed widely in vascular endothelium, cardiovascular tissue, renal tissue, and intestinal epithelia, leading to the systemic beyond pneumonia manifestations of the disease [6].

Pneumonia, an almost “sine qua non” of hospitalized patients due to COVID-19, presents an extended clinical spectrum from asymptomatic to severe/critical ARDS with multiorgan failure. Individuals with COVID-19 pneumonia are categorized as having severe illness if they exhibit SpO_2_ < 94% on room air at sea level, a PaO_2_/FiO_2_ ratio < 300 mmHg, a respiratory rate exceeding 30 breaths/min, and/or infiltrates exceeding 50% of the lung area [7]. Critical illness defines ARDS requiring mechanical ventilation, septic shock, and/or multiple organ dysfunction [7,8].

The eventual occurrence of long-term lung sequelae after the resolution of any severity manifestations, including severe/critical pneumonia, in COVID-19 patients has been of interest in previous studies. This especially regards the potential of SARS-CoV-2 to induce widespread lung fibrosis as well as the optimal early preventive and/or long-term appropriate treatment. The majority of post-COVID-19 follow-up studies lack comprehensive documentation of post-discharge therapeutic interventions, with limited research specifically assessing the efficacy of corticosteroids, immunomodulators, or antifibrotic agents in addressing long-term pulmonary sequelae [9,10,11,12,13,14]. Furthermore, to the best of our knowledge, no data are available regarding the “non-interventional” approach in survivors of severe/critical pneumonia in COVID-19 after hospital discharge.

This study aimed to investigate the “non-interventional” approach in the long term, e.g., the occurrence of lung fibrotic sequelae in survivors of severe/critical pneumonia in COVID-19 after hospital discharge without further administration of corticosteroids, immunomodulators, or antifibrotics.

## 2. Materials and Methods

### 2.1. Study Design

This is a prospective, follow-up study of severe/critical COVID-19 pneumonia survivors discharged from General University Hospital “Attikon”. In order to evaluate the long-term lung fibrosis sequelae, patients hospitalized from March 2020 to October 2022 with severe/critical COVID-19 pneumonia and re-evaluated from 3 to 12 months post-discharge were included in this study.

Individuals with COVID-19 pneumonia were categorized as having severe illness if they exhibited SpO_2_ < 94% on room air at sea level, a PaO_2_/FiO_2_ ratio < 300 mm Hg, a respiratory rate exceeding 30 breaths/min, and/or infiltrates exceeding 50% of lung area [7]. Affected by critical illness were those on ARDS requiring mechanical ventilation, septic shock, and/or multiple organ dysfunction [7,8].

Written informed consent was obtained from each participant. This study was approved by the Institutional Ethics Committee of the General University Hospital “Attikon” (ID: 487/3 September 2020).

### 2.2. Patients’ Parameters

In all patients, demographics, comorbidities, and clinical data were recorded. Patients were assessed at 3, 6, and 12 months after discharge. Pulmonary function tests, including forced expiratory volume in 1 s (FEV1), forced vital capacity (FVC), diffusing lung capacity for carbon monoxide (DLCO); chest high resolution computed tomography (HRCT) scan; modified Medical Research Council (mMRC) dyspnea scale; and St. George’s Respiratory Questionnaire (SGRQ) were evaluated [15,16].

Upon admission to the hospital, all patients were submitted to chest CT, HRCT, or CT pulmonary angiography (CTPA) in cases of suspected pulmonary embolism. All patients were re-evaluated with a chest HRCT scan in the supine position during deep inspiration in every follow-up assessment. The CT scanner used for all examinations was a Philips Brilliance 64-slice machine (Philips Healthcare, Amsterdam, The Netherlands). Scans were performed using a spiral technique of 1 mm collimation with a 1 mm interslice gap. Images were reconstructed using advanced iterative algorithms (iDose 4) at both soft-tissue (width 350 HU; level 50 HU) and lung parenchymal (width 1500 HU; level −700 HU) window settings.

**Figure 1:**
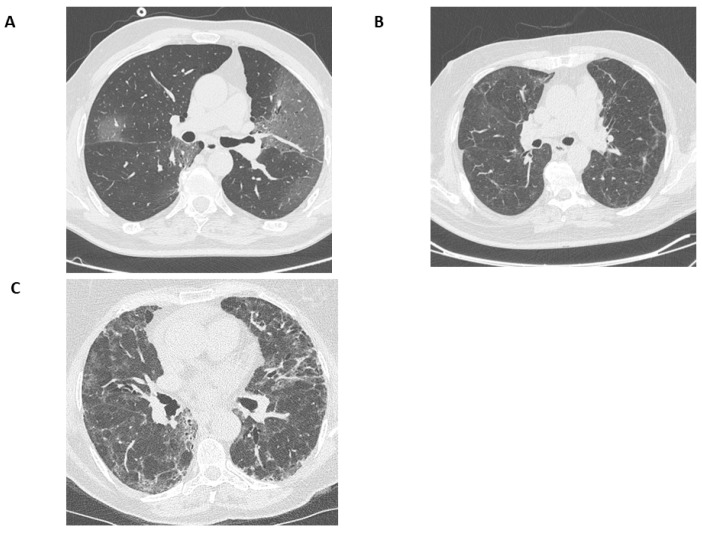
The radiological patterns assessed during the evaluation of follow-up HRCT after hospital discharge: (**A**) COVID-19-associated pulmonary opacities, (**B**) parenchymal bands, and (**C**) fibrotic-like changes. HRCT = high resolution computed tomography.

Three thoracic radiologists (with 3, 6, and 25 years of experience, respectively) blinded to the clinical data interpreted the CT scans independently. Any disagreement was resolved by discussion to reach a consensus. The initial CT scan performed upon admission was compared to the subsequent scans regarding the following parameters: (a) COVID-19 pneumonia-associated pulmonary opacities (ground glass) and the extent of them is shown in one patient included in this study (Figure 1A); (b) parenchymal bands in Figure 1B; (c) fibrotic-like changes such as a non-specific interstitial pneumonia (NSIP) pattern in Figure 1C; and (d) a usual interstitial pneumonia (UIP) pattern [17,18].

The quantification of the total extent of pulmonary parenchyma affected by COVID-19-associated pulmonary opacities was held with the “Co.V.A.Sc.” (COVID-19 Visual Assessment Scale) [19,20]. The “Co.V.A.Sc.” (COVID-19 Visual Assessment Scale) [13] is a percentage-based scoring system designed to assist observers in evaluating chest CT scans of RT-PCR-confirmed COVID-19 patients, with published data suggesting that it may also contribute to risk assessment in these patients [20]. It enables the approximate estimation and quantification of the overall extent of pulmonary parenchyma affected by COVID-19-related opacities. These opacities, as defined by the Fleischner Society Glossary of Terms for Thoracic Imaging, are regions that appear more opaque compared to adjacent areas due to X-ray beam attenuation [18].

In December 2023, a conclusive assessment was conducted either by accessing the electronic files of each patient and/or through personal communication via phone calls recording the vital status (deceased or alive), the dyspnoea symptoms using the mMRC scale, and their performance status (if they have returned to their pre-COVID-19 level of activity).

### 2.3. Statistics

Continuous variables were expressed as medians (25–75th interquartile range, IQR) and compared using Mann–Whitney U test, Kruskal–Wallis test, or repeated-measures ANOVA if appropriate. Categorical variables, whenever dichotomous or nominal, were expressed as *n* (%) and compared by the χ^2^ test, Wilcoxon, or Friedman test, where appropriate. Univariate and multivariate regression analyses were performed to evaluate the influence of demographic and clinical data on the presence of fibrotic-like changes during follow-up. All data analyses were performed in SPSS (version 26.0), and *p*-values less than 0.05 were considered statistically significant. Patients with missing data were not included in the analysis.

## 3. Results

### 3.1. Study Population

**Figure 2:**
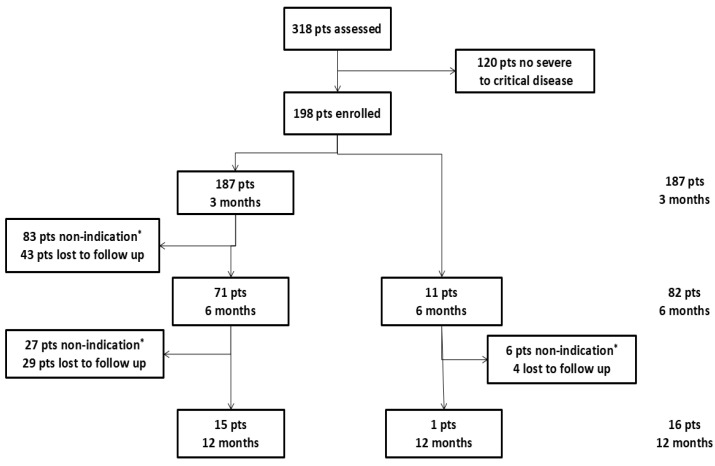
Study population flowchart. pts = patients * No indication for follow-up was defined when the following criteria were met: forced vital capacity (FVC) > 80%, modified Medical Research Council dyspnea scale (mMRC) = 0, and extent of COVID-19-related opacities < 25%.

From March 2020 to October 2022, more than 6000 patients were hospitalized at our hospital with COVID-19 of any severity, and 318 opted to be evaluated at the COVID-19 follow-up dedicated outpatient clinic of our hospital. Out of 318 patients, 198 were eligible for this study and were further analyzed (Figure 2). Moreover, 121 patients were males (61.1%), 100 (50.5%) were ever smokers, with a median (IQR) age of 57 years (49–66) and a median BMI of 29.4 kg/m^2^ (27.3–33.1). Moreover, 152 (77.8%) had negative vaccination history against SARS-CoV-2. The median length of hospitalization was 17 days (12–25). The demographic and medical characteristics of the study population are shown in Table 1.

During this study, 131 (66.1%) patients were admitted to the hospital in the pre-delta period, 48 (24.2%) in the delta-variant prominent period and 19 (9.5%) in the omicron-variant prominent period based on the Greek epidemiological data [21].

Moreover, 187 (94.4%) patients were assessed at 3 months, 82 (41.1%) at 6 months, and 16 (8%) at 12 months (Figure 2). The functional and radiological characteristics of patients lost to follow-up at each time point are shown in Table A1. Patients lost to follow-up had a better clinical status at the 3-month visit compared to those lost to follow-up afterwards (Table A1).

### 3.2. Therapy

During hospitalization, 184 (92.9%) patients received remdesivir, 189 (95.4%) dexamethasone, and 105 (53%) received other immunomodulatory treatment indicated for SARS-CoV-2 infection based on the National and International Guidelines (Table 1) [7]. Depending on the period of hospitalization according to guidelines of that time, a number of patients received medications, such as hydroxychloroquine and macrolides, which are no longer indicated for COVID-19. After discharge from the hospital, none of the study patients received steroids, immunomodulatory drugs, or antifibrotic therapy.

### 3.3. CT Findings

**Figure A1:**
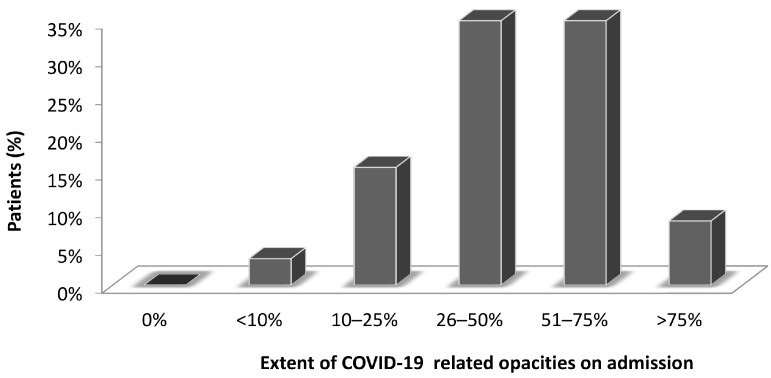
The extent of the COVID-19-associated pulmonary opacities on admission chest CT; 7 (3.5%) patients presented with <10%, 31 (10.6%) with 10–25%, 72 (36.3%) with 26–50%, 71 (35.8%) with 51–75%, and 17 (8.5%) with >75% extent. CT = computed tomography.

The evaluation of admission chest CT demonstrated that, according to the extent of the COVID-19-associated pulmonary opacities, 7 (3.5%) patients presented with less than 10% extent, 31 (10.6%) patients with 10–25% extent, 72 (36.3%) patients with 26–50% extent, 71 (35.8%) patients with 51–75% extent, and 17 (8.5%) patients with more than 75% extent (Figure A1). Two (1%) patients had pre-existing fibrotic changes (fibrotic NSIP pattern).

**Figure A2:**
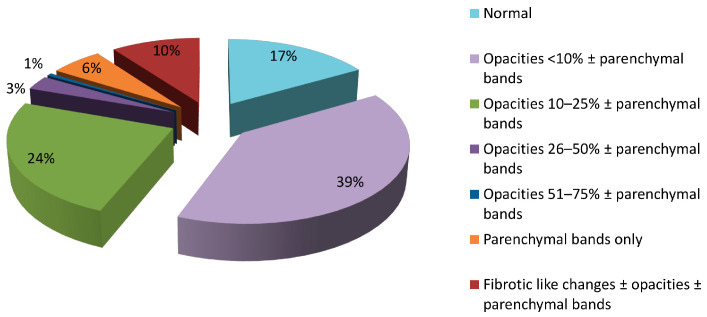
HRCT findings at 3 months after hospital discharge (*n* = 168). HRCT = high-resolution computed tomography.

**Figure A3:**
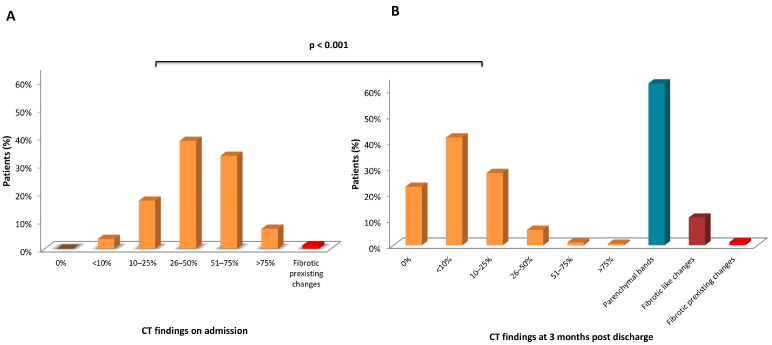
CT findings (**A**) on admission and (**B**) at 3 months after hospital discharge (*n* = 168). At 3 months, 140/168 (83.3%) had residual findings; 70 (41.6%) had < 10%, 47 (27.9%) had 10–25%, 10 (5.9%) had 26–50%, and 3 (1.7%) had 51–75% extent of the COVID-19-associated pulmonary opacities. Moreover, 105 (62.5%) had parenchymal lines and 17 (10.1%) fibrotic-like changes. There was a statistically significant reduction in the extent of the opacities at 3 months (*p* < 0.001). CT = computed tomography.

At 3 months after discharge, 140/168 (83.3%) patients had residual findings; 70 (41.6%) presented with less than 10% extent, 47 (27.9%) patients with 10–25% extent, 10 (5.9%) patients with 26–50% extent, and 3 (1.7%) patients with 51–75% extent of the COVID-19-associated pulmonary opacities. Moreover, 105 (62.5%) had parenchymal lines and 17 (10.1%) fibrotic-like changes (Figure A2). There was a statistically significant reduction in the extent of the opacities at 3 months compared to the admission CT for both patients with and without fibrotic-like changes (r = 0.6, *p* < 0.001) (Figure A3).

**Figure 3:**
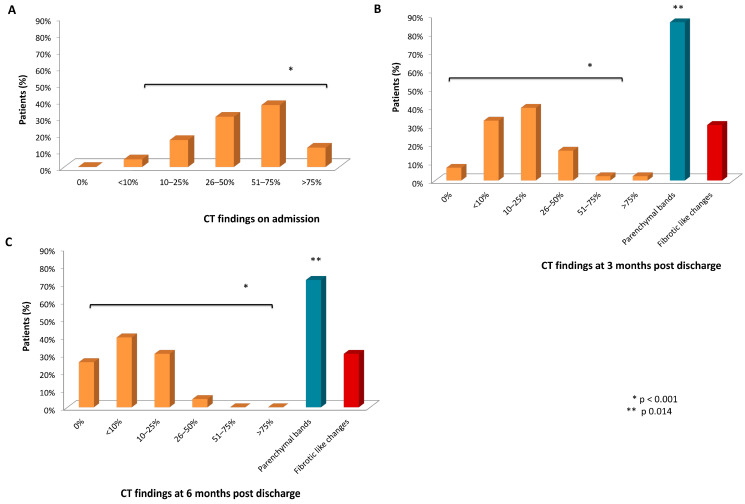
CT findings during follow-up: (**A**) on admission, (**B**) at 3 months after hospital discharge, and (**C**) at 6 months (*n* = 43). At 6 months, 36/43 (83.7%) had residual findings: 17 (39.5%) < 10%, 13 (30.2%) 10–25%, 2 (4.6%) 26–50%, and 0 (0%) > 50% extent of the COVID-19-associated pulmonary opacities. Moreover, 31 patients (72.1%) had parenchymal lines and 13 (30.2%) had fibrotic-like changes. There was a statistically significant reduction in the extent of the opacities (*p* < 0.001) and in the presence of parenchymal bands at each time point (*p* = 0.014). CT = computed tomography.

Forty-three patients (thirteen with fibrotic-like changes) had two consecutive CTs at 3 and 6 months, and five patients (four with fibrotic-like changes) had three consecutive CTs at 3, 6, and 12 months. There was a statistically significant reduction in the extent of the opacities (Kendall’s w = 0.616, *p* < 0.001 and Kendall’s w = 0.957, *p* = 0.002, respectively) and in the presence of parenchymal bands at each time point (r = 0.26, *p* = 0.014 and Kendall’s w = 0.6, *p* = 0.025, respectively) (Figure 3).

Eighteen (9.1%) patients had fibrotic-like changes on the follow-up CT scans. Two patients with findings mainly in the apices, fourteen patients with a fibrotic NSIP pattern, and two patients with an NSIP pattern—minimal findings. No one exhibited a typical UIP pattern. A statistically significant reduction in the extent of opacities and the presence of parenchymal bands was observed at each time point. Additionally, there was a qualitative elimination of fibrotic-like changes. Finally, in the two patients where preexisting fibrotic changes were identified on admission, no worsening of fibrotic changes were observed during follow-up.

The demographic and medical characteristics of patients with fibrotic-like changes are shown in Table 1. Τhere was no statistically significant difference between patients with fibrotic-like changes and patients without fibrotic-like changes regarding demographics and the treatment received during hospitalization. There was no statistically significant difference in comorbidities except for dyslipidemia (30.5% in the group without fibrotic-like changes vs. 5.5% with fibrotic-like changes, *p* = 0.025). However, a statistically significant difference was observed in the length of hospital stay (16 (12–22) in the group without fibrotic-like changes vs. 28.5 (18–64) with fibrotic-like changes, *p* = 0.001) and the rate of intubation during hospitalization (13.8% in the group without fibrotic-like changes vs. 33.3% with fibrotic-like changes, *p* = 0.003) between the two groups. According to the multivariate logistic regression analysis, only length of stay (OR 1.057, 95%CI 1.035–1.088; *p* = 0.01) was correlated with the presence of fibrotic-like changes (Table A2).

### 3.4. Spirometry

Spirometry was performed in 172 (85.8%) patients at 3 months, in 66 (33.3%) at 6 months, and in 12 (60.6%) at 12 months.

**Figure 4:**
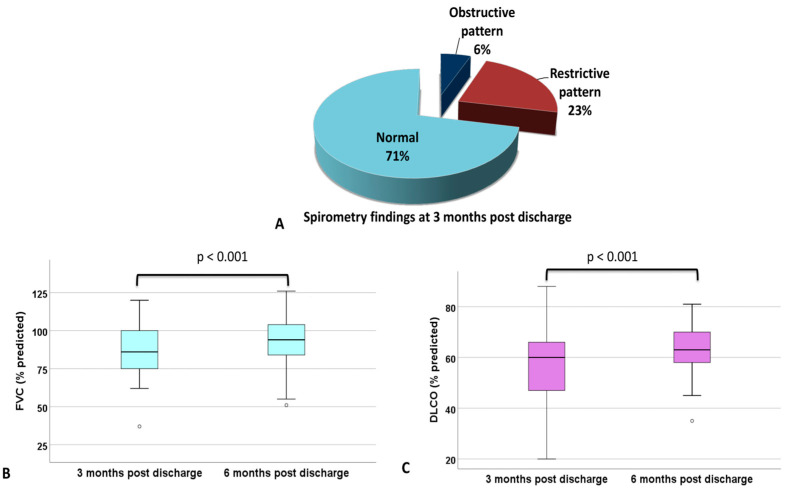
Lung function tests. (**A**) Spirometry patterns at 3 months after hospital discharge (*n* = 172), (**B**) FVC change at 3 and 6 months after hospital discharge (*n* = 55). (**C**) DLCO alteration at 3 and 6 months after hospital discharge (*n* = 29). There was a statistically significant improvement in FVC (*p* < 0.001) and DLCO (*p* < 0.001). FVC = forced vital capacity, DLCO = lung diffusing capacity for carbon monoxide.

At 3 months after discharge, 123/172 (71.5%) had normal spirometry test results, 10/172 (5.8%) had an obstructive pattern, and 39/172 (22.7%) had a restrictive pattern (Figure 4). The median FEV1 was 93% of predicted (80–100%), and FVC was 93% (80–100%). Patients exhibiting fibrotic-like changes demonstrated statistically significant lower FVC values at 3 and 6 months compared to those without such changes (68% (62–84%) vs. 92.5% (82–103%) at 3 months, r = 0.32, *p* < 0.001), (86% (61–98%) vs. 94 (86.5–100%) at 6 months, r = 0.264, *p* = 0.035) (Table 2).

Fifty-five patients (twelve with fibrotic-like changes) had consecutive spirometry at 3 and 6 months and six patients (three with fibrotic-like changes) had consecutive spirometry at 3, 6, and 12 months. There was a statistically significant improvement in FEV1 (partial η^2^ = 0.49, *p* < 0.001 and partial η^2^ = 0.71, *p* = 0.01, respectively) and FVC (partial η^2^ = 0.51, *p* < 0.001 and partial η^2^ = 0.74, *p* = 0.006, respectively) with relative median increase in FVC 6% (2–11.6%) at 6 months and 29% (14.8–44.2%) at 12 months (Figure 4).

**Figure 5:**
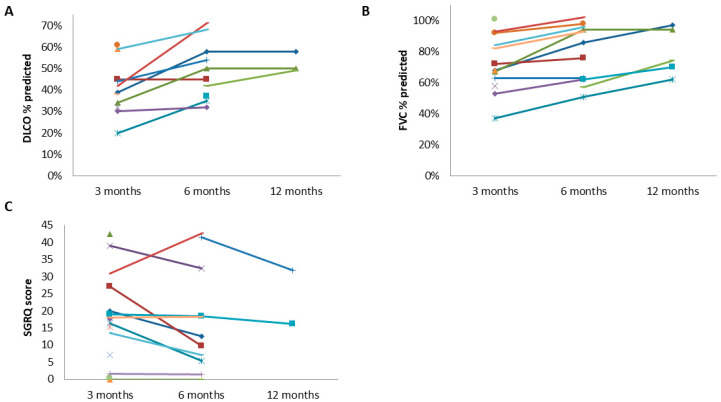
Changes in (**A**) FVC, (**B**) DLCO, and (**C**) SGRQ score at 3, 6, and 12 months after hospital discharge in patients with fibrotic-like changes (*n* = 18). FVC = forced vital capacity, DLCO = lung diffusing capacity for carbon monoxide, SGRQ = St. George’s Respiratory Questionnaire.

The FVC changes in patients with fibrotic-like changes are shown in Figure 5.

### 3.5. Diffusion Capacity

Diffusion capacity was measured in 122 (61.6%) patients at 3 months, in 53 (26.8%) at 6 months, and in 10 (5.1%) at 12 months. The fact that fewer patients underwent measurement of diffusing capacity compared to spirometry is attributed to technical issues in the laboratory during that period.

At 3 months, DLCO ≥ 70% was observed in 47/122 (38.5%) patients. The median DLCO was 65% (58–78.2%) at 3 months. Patients with fibrotic-like changes exhibit statistically significant lower DLCO values at 3 and 6 months compared to patients without fibrotic-like changes (39% (34–59%) vs. 66% (60–78%) at 3 months, r = 0.38, *p* < 0.001), (57.5% (45–68%) vs. 66% (59.5–76%) at 6 months, r = 0.29, *p* = 0.031) (Table 2).

Twenty-nine patients (seven with fibrotic-like changes) had consecutive measurements of DLCO at 3 and 6 months, and three patients (two with fibrotic-like changes) had consecutive measurements of DLCO at 3, 6, and 12 months. There was a statistically significant improvement in DLCO (partial η^2^ = 0.401, *p* < 0.001, and partial η^2^ = 0.991, *p* < 0.001, respectively) with a relative median increase of 9.4% (1.4–14%) at 6 months and 47% (43.3–47.9%) at 12 months (Figure 4).

The DLCO alteration in patients with fibrotic-like changes is shown in Figure 5.

### 3.6. Oxygen Therapy and SpO_2_

At discharge, 42 (21.2%) patients required oxygen therapy, 9 (4.5%) at 3 months, 1 (0.5%) at 6 months, and no patients at 12 months (*p* < 0.001). The median SpO_2_ was 98% (97–98%) at 3 months, 98% (97–98%) at 6 months, and 98% (98–98%) at 12 months.

### 3.7. mMRC Scale

**Figure A4:**
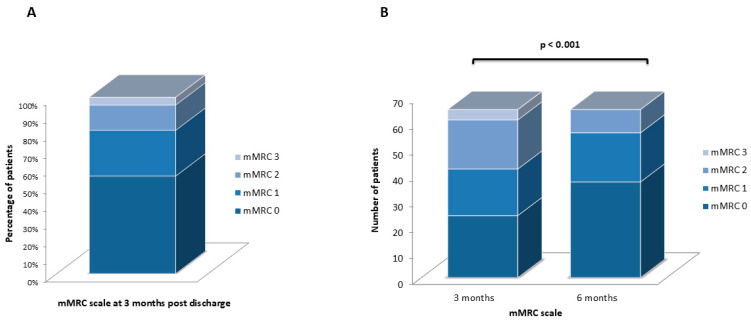
mMRC scale (**A**) at 3 months after hospital discharge (*n* = 181) and (**B**) at 3 and 6 months after hospital discharge (*n* = 65). At 6 months 37/65 (57%) had a mMRC scale of 0, 19 (29.2%) had a mMRC of 1, and 9 (13.8%) had a mMRC of 2. The number of patients with various levels of dyspnea symptoms significantly reduced at 6 months (*p* < 0.001). mMRC = modified Medical Research Council dyspnea scale.

At 3 months, 181 (91.4%) patients were assessed with the mMRC scale; at 6 months, 74 (37.4%) patients were assessed; and at 12 months, 14 (7%) patients were assessed. At 3 months, 100/181 (55.2%) had a mMRC scale of 0; 47 (25.9%) patients had a mMRC of 1; 26 (14.3%) patients had a mMRC of 2; and 8 (4.4%) patients had a mMRC of 3 (Figure A4). Patients with fibrotic-like changes had higher mMRC scale, compared to patients without fibrotic-like changes at 3 and 6 months, with a statistically significant difference at 3 months (ω = 0.26, *p* = 0.005) (Table 2).

Sixty-five patients (twelve with fibrotic-like changes) had consecutive assessments with the mMRC scale at 3 and 6 months, and nine patients (four with fibrotic-like changes) had consecutive assessments at 3, 6, and 12 months. The number of patients with various levels of dyspnea symptoms significantly reduced at each time point (r = 0.46, *p* < 0.001, and Kendall’s w = 0.722, *p* = 0.002, respectively) (Figure A4).

### 3.8. SGRQ

At 3 months, 170 (85.6%) patients completed the SGRQ; at 6 months, 66 (33.3%) patients completed the SGRQ; and at 12 months, 7 (3.5%) patients completed the SGRQ. At 3 months the median SGRQ was 11.65 (0–24.3). Patients with fibrotic-like changes had higher SGRQ scores at 3 and 6 months compared to those without fibrotic-like changes, but without statistically significant differences (Table 2).

Fifty-seven patients (ten with fibrotic-like changes) completed the SGRQ at 3 and 6 months with a statistically significant difference (partial η^2^ = 0.084, *p* = 0.027). Consecutive evaluations at 3, 6, and 12 months were performed in two patients (one with fibrotic-like changes). The variation of SGRQ in patients with fibrotic-like changes is shown in Figure 5.

### 3.9. Conclusive Evaluation

Following the conclusive evaluation of patients in December 2023, 193/198 patients (97.5%) were confirmed to be alive, one patient was deceased, and data for four patients were missing (not included in the national data recording system due to different nationalities). Notably, all patients with fibrotic-like changes were alive.

Telephonic communication has been established with 113/198 (57%) patients; the remaining individuals were either not located or declined to respond. Among the patients who underwent telephonic communication, 91/113 (80.5%) individuals had mMRC 0, while 101/113 (89.3%) patients mentioned that they have returned to their pre-illness level of activity. In the subgroup of patients with fibrotic-like changes, telephonic communication was established with all of them. Among these patients, sixteen (88.9%) had an mMRC scale of 0, and two (11.1%) had an mMRC scale of 1. Importantly, all patients with fibrotic-like changes reported a return to their pre-COVID-19 level of activity.

## 4. Discussion

This prospective study, including only survivors of severe/critical COVID-19 pneumonia, documented the significant improvement of all imaging, functional, and clinical parameters by applying the “non-interventional” approach, i.e., without further administration after hospital discharge of corticosteroids, immunomodulators, or antifibrotics.

To our knowledge, this is the first study that examines the evolution of severe/critical COVID-19 pneumonia by applying the “non-interventional” approach after hospital discharge. The majority of follow-up studies after COVID-19 infection do not clarify what treatment patients received after discharge, while there are a few studies that have looked at the utility of steroids and antifibrotic drugs in patients with residual respiratory, functional, and/or imaging sequelae [9,10,11,12,13,14].

The beneficial effect of corticosteroids in post-COVID-19 patients is still uncertain as a result of the heterogeneity of existing studies in terms of design and patient selection, but mainly due to the lack of placebo-controlled studies. Yüksel et al. conducted a study showing that patients with “post-COVID-19 ILD”, defined as the presence of respiratory symptoms, hypoxemia, restrictive lung function, and residual parenchymal infiltrates on thorax HRCT at hospital discharge, who received a four-week course of steroids after discharge (*n* = 135) had higher rates of good clinical response (mMRC 0) and functional response (FVC > 80%) compared to the control group (*n* = 127) (62.9% vs. 33.3%; 54.1% vs. 33.7%, *p* < 0.001, respectively) [9]. Although the steroid group also tended to have a higher radiological response than the control group, this difference was not statistically significant (61.1% vs. 51.1%, *p* = 0.159). Myall K.J. et al. explored the stimulatory impact of steroids on patients exhibiting residual functional and imaging findings categorized as “post-COVID-19 ILD”, predominantly organizing pneumonia pattern, with 30 patients having received steroid treatment resulting in a mean relative increase in DLCO post-treatment of 31.6% (standard deviation [SD] ± 27.6, *p* < 0.001) and FVC of 9.6% (SD ± 13.0, *p* = 0.014) with significant symptomatic and radiological improvement [10]. On the other hand, the COLDSTER trial is a single-center, open-label, parallel-group, randomized trial including patients with persisting dyspnea, resting hypoxemia, or exertional SaO_2_ desaturation and diffuse abnormalities involving ≥20% of lung parenchyma on HRCT, 3–8 weeks after the onset of acute COVID-19 symptoms. Participants were randomly assigned to receive either high-dose prednisolone (40 mg/day for 1 week, followed by 30 mg/day for 1 week, 20 mg/day for 2 weeks, and 10 mg/day for 2 weeks) or low-dose prednisolone (10 mg/day for the entire 6 weeks) [11]. This study showed no significant difference between the two groups in clinical, radiological, physiological, and quality-of-life outcomes, suggesting the lack of justification for the administration of high doses of steroids in post-COVID-19 patients.

These findings cannot be easily compared to the results of our own study due to methodological discrepancies. For example, although in the study by Myall K.J. [10], the epidemiological characteristics of the patients and the duration of hospitalization align with the data from our study, a smaller proportion of patients (17.2%) received steroids during the acute phase, while a larger proportion (45.6%) required invasive mechanical ventilation. Notable, no data on imaging findings during hospitalization were provided. Additionally, patients diagnosed with “post-COVID-19 ILD” exhibited significantly higher FVC % predicted and DLCO % predicted values compared to patients in our study with fibrotic-like changes, further highlighting the divergent clinical assessment of patients with the so-called “post-COVID-19 ILD”. In the COLDSTER trial [11], although the epidemiological characteristics are comparable with our study patients, with 98% having severe or critical disease and 43% receiving invasive mechanical ventilation or high-flow nasal oxygen therapy, data on radiological findings and functional parameters before treatment are lacking. Finally, Yüksel et al. presented the data from a multicenter, prospective study (STERCOV-ILD) [9]; however, epidemiological and severity data during hospitalization were not reported. The key difference from our study is that the persistent infiltrates in post-COVID-19 patients were defined as post-COVID-19 ILD findings by the authors [9].

In this study, we categorized radiological sequelae at follow-up into infiltrates, parenchymal bands, and “fibrotic-like” changes. The term “fibrotic-like” changes was specifically applied to post-COVID-19 patients, as the majority of these findings were limited, coinciding with clinical and functional improvement [22]. It is worth noting that the term “post-COVID-19 ILD” in various publications, including the studies mentioned earlier, encompasses both “fibrotic-like” changes and persistent infiltrates considered as an organizing pneumonia component. In our study, post-COVID-19 infiltrates were limited and/or completely regressed without the need for corticosteroids or other interventions, highlighting the favorable, post-hospital discharge, natural course of severe/critical COVID-19 pneumonia. Furthermore, 18 (9.1%) patients exhibited “fibrotic-like” changes during the follow-up period. Despite the consideration of the severity of the initial disease as a prognostic factor for post-COVID-19 fibrosis, there is a notable variation in the incidence of fibrosis after COVID-19 across different studies, with some studies indicating much higher rates up to 35–70% [23,24]. The inconsistency in these findings can be attributed to the heterogeneity in study designs and the absence of clear criteria for classifying fibrosis after COVID-19 pneumonia, as well as the lack of consensus on the optimal assessment time following the acute phase.

Post-COVID-19 residual opacities and fibrotic-like changes may be associated with extensive lung involvement during the acute episode, ARDS, and/or the use of mechanical ventilation in intubated patients [22]. Similar findings have been reported in patients recovering from ARDS of various etiologies, particularly ARDS caused by viral infections such as Influenza A. Notably, in cases of ARDS due to Influenza A, studies have demonstrated that respiratory function abnormalities and imaging findings, even in the presence of fibrotic-like changes, can improve or even resolve within a year without any intervention [25]. The pathophysiology of any ARDS is multifactorial and includes inflammation, barrier disruption, interstitial and airspace edema, cell injury, and cell death [26]. Diffuse alveolar damage is the pathologic hallmark of ARDS. No matter how complex the pathophysiologic mechanisms of ARDS are, repair of the alveolar epithelium may occur and is regulated by crosstalk between multiple alveolar cell types and the extracellular matrix [27]. Management of ARDS focuses mainly on the treatment of the triggering infection, respiratory support, careful fluid management, and the best supportive measures.

There are limited data from previous studies on the effectiveness of antifibrotic agents (nintedanib and pirfenidone) in the prevention of post-COVID-19 fibrosis. The existing data are derived from individual cases or case series [12,13,14] with a lack of comparability to our study. In clinical practice, the use of antifibrotic drugs in these patients is predominantly based on empirical treatment, and further clinical studies are crucial. We await the results of ongoing randomized, double-blind interventional studies to provide insights into the efficacy of antifibrotic agents in patients with findings indicative of post-COVID-19 fibrosis [ClinicalTrials.gov]. However, in our study, patients with fibrotic-like changes had a clear clinical and functional improvement with concurrently stable or improved imaging findings, eliminating the potential need for specific treatment.

There are some limitations to this study. This is a single-center trial with a moderate number of patients upon initial evaluation but with low follow-up rates, particularly at 12 months, not permitting generalization of results. Due to technical issues, pulmonary and functional assessment was not feasible at 12 months for the entire cohort of our study patients. However, our data referring to a well-characterized population of severe COVID-19 patients in a tertiary hospital are indicative of daily clinical practice and provide original evidence of long-term follow-up without any therapeutic intervention after hospital discharge.

## 5. Conclusions

This prospective study, including only survivors of severe/critical COVID-19 pneumonia, documented the significant improvement of all imaging, functional, and clinical parameters by applying the “non-interventional” approach, i.e., without further administration after hospital discharge of corticosteroids, immunomodulators, or antifibrotics. These data do not advocate any post-COVID-19 severe/critical pneumonia “epidemic of widespread pulmonary fibrosis”.

## Figures and Tables

**Table 1:** The epidemiological and clinical characteristics of the study population during hospitalization (*n* = 198) *.

	All Patients	Patients Without Fibrotic-like Changes	Patients With Fibrotic-like Changes	*p*-Value
Number of pts	198	180 (90.9)	18 (9.1)	
Age (years), median (IQR)	57 (49–66)	57 (48–66)	57 (52–67)	0.69
Sex (male), *n* (%)	121 (61.1)	119 (66.1)	12 (66.6)	0.612
Smoking history, *n* (%)	100 (50.5)	89 (49.4)	11 (61.1)	0.345
ΒΜΙ (kg/m^2^), median (IQR)	29.4 (27.3–33.1)	29.4 (27.4–33)	29.1 (26.8–32.3)	0.414
Vaccinated, *n* (%)	46 (23.2)	43 (23.8)	3 (16.6)	0.481
Comorbidities				
COPD/asthma, *n* (%)	13 (6.5)	10 (5.5)	3 (16.6)	0.483
ILD, *n* (%)	2 (1)	2 (1.1)	0 (0)	N/A
Diabetes, *n* (%)	35 (17.6)	34 (18.8)	1 (5.5)	0.155
Dyslipidemia, *n* (%)	56 (28.3)	55 (30.5)	1 (5.5)	0.025
Hypertension, *n* (%)	79 (39.8)	73 (40.5)	6 (33.3)	0.551
CVD/Heart failure, *n* (%)	12 (6.1)	12 (6.6)	0 (0)	N/A
Autoimmune disease, *n* (%)	17 (8.6)	16 (8.8)	1 (5.5)	0.63
Current cancer, *n* (%)	3 (1.5)	3 (1.6)	0 (0)	N/A
Length of stay (days), median (IQR)	17 (12–25)	16 (12–22)	28.5 (18–64)	0.001
COVID-19 therapy ^#^, *n* (%)				
Remdesivir	184 (92.9)	166 (92.2)	18 (100)	0.22
Dexamethasone	189 (95.4)	171 (95)	18 (100)	0.332
Immunomodulators	105 (53)	83 (46.1)	12 (66.6)	0.224
Respiratory support				
FiO_2_ ≥ 50%, *n* (%)	66 (37.3)	62 (34.4)	4 (22.2)	0.294
HFNC, *n* (%)	92 (46.4)	84 (46.6)	8 (44.4)	0.857
Intubation, *n* (%)	31 (15.6)	25 (13.8)	6 (33.3)	0.003

CVD = cardiovascular disease, FiO_2_ = fraction of inspired oxygen, HFNC = high-flow nasal cannula therapy. * Functional parameters not performed during hospitalization. ^#^ Based on National and International Guidelines. N/A: Not Applicable.

**Table 2:** Epidemiological, clinical, and functional parameters of the study population based on the presence of fibrotic-like changes at 3 and 6 months after hospital discharge.

	Patients With Fibrotic-like Changes at 3 Months	Patients Without Fibrotic-like Changesat 3 Months	*p*-Value	Patients With Fibrotic-like Changes at 6 Months	Patients Without Fibrotic-like Changes at 6 Months	*p*-Value
Number of patients	17	170		14	68	
Age (years), median [IQR]	58 (52–67)	57 (48–66)	0.696	56 (49–66)	61 (53–67)	0.386
Sex (male), *n* (%)	11 (64.7)	104 (61.2)	0.776	8 (57.1)	37 (54.4)	0.988
Functional parameters						
FEV1 (%), median (IQR)	68 (63–86)	91.5 (80–102)	<0.001	88 (65–100)	94 (84–106)	0.151
FVC (%), median (IQR)	68 (62–84)	92.5 (82–103)	<0.001	86 (61–98)	94 (86.5–100)	0.035
DLCO (%), median (IQR)	39 (34–59)	66 (60–78)	<0.001	57.5 (45–68)	66 (59.5–76)	0.031
SpO_2_ (%), median (IQR)	98 (97–98)	98 (97–98)	0.233	98 (97–98)	98 (97–98)	0.143
mMRC scale,			0.005			0.554
*n* (%)						
0	6 (35.3)	94 (55.3)		7 (50)	34 (50)	
1	2 (11.7)	45 (26.5)		3 (21.4)	20 (29.4)	
2	7 (41.2)	19 (11.2)		3 (21.4)	6 (8.8)	
3	1 (5.9)	3 (1.7)		0 (0)	1 (1.5)	
NA	1 (5.9)	9 (5.3)		1 (7.2)	7 (10.3)	
SGRQ, median (IQR)	16.9 (4.4–23.9)	10.8 (0–24.3)	0.377	28.1 (6.23–32.9)	14.9 (1.1–27.8)	0.24

FEV1 = forced expiratory volume in 1 s, FVC = forced vital capacity, DLCO = lung diffusing capacity for carbon monoxide, SpO_2_ = pulse oxygen saturation, mMRC = modified Medical Research Council dyspnea scale, SGRQ = St. George’s Respiratory Questionnaire.

## Data Availability

Data are contained within the article and Appendix A.

## Appendix A

**Table A1:** Clinical, functional, and imaging parameters of the study population based on those lost to follow-up at 6 and 12 months after hospital discharge.

	Parameters at 3 Months			Parameters at 6 Months		
	Patients Follow-up at 6 Months	Patients Lost to Follow-up at 6 Months	*p*-Value	Patients Follow-up at 12 Months	Patients Lost to Follow-up at 12 Months	*p*-Value
Number of patients	71	126		16	66	
Age (years), median (IQR)	58 (50–67)	56 (48–66)	0.383	57.5 (48.5–66)	56 (50–66.5)	0.908
Sex (male), *n* (%)	40 (56.3)	81 (66.3)	0.271	8 (50)	40 (60.6)	0.439
Functional parameters						
FEV1 (%), median (IQR)	86 (74–100)	93 (83–102)	0.01	93 (80–97)	94 (83–106)	0.204
FVC (%), median (IQR)	85 (74–100)	94 (84–103)	0.011	91 (84–95)	97 (84–105)	0.107
DLCO (%), median (IQR)	60.5 (44.5–65.5)	70 (61–80)	<0.001	59 (51.5–50)	68 (59–77)	0.006
SpO_2_ (%), median (IQR)	98 (97–98)	98 (97–98)	0.931	98 (97–98)	98 (97–98)	0.987
mMRC scale,			<0.001			0.294
*n* (%)						
0	26 (36.6)	74 (58.7)		7 (43.8)	34 (51.5)	
1	19 (26.8)	28 (22.2)		8 (50)	15 (22.8)	
2	19 (26.8)	7 (5.6)		1 (6.2)	8 (12.1)	
3	5 (7)	3 (2.4)		0 (0)	1 (1.5)	
NA	2 (2.8)	14 (11.1)		0 (0)	8 (12.1)	
SGRQ, median (IQR)	19.9 (4.4–34.8)	6.4 (0–17.6)	<0.001	28.4 (13.6–32.2)	8 (0.3–26.5)	0.038
Imaging parameters						
Number of patients	43	125		14	37	
COVID-19 opacities (Co.VA.Sc score), *n* (%)			<0.001			0.201
0	3 (6.9)	35 (28)		2 (14.3)	10 (27)	
1	14 (32.5)	56 (44.8)		5 (35.7)	17 (46)	
2	17 (39.5)	30 (24)		4 (28.6)	9 (24.3)	
3	7 (16.2)	3 (2.4)		2 (14.3)	1 (2.7)	
4	1 (2.3)	1 (0.8)		1 (7.1)	0 (0)	
5	1 (2.3)	0 (0)		0 (0)	0 (0)	
Parenchymal bands, *n* (%)	37 (86)	68 (54.4)	<0.001	12 (85.7)	24 (64.9)	0.145
Fibrotic-like changes, *n* (%)	13 (30.2)	4 (3.3)	<0.001	4 (28.6)	8 (21.6)	0.602

FEV1 = forced expiratory volume in 1 s, FVC = forced vital capacity, DLCO = lung diffusing capacity for carbon monoxide, SpO_2_ = pulse oxygen saturation, mMRC = modified Medical Research Council dyspnea scale, SGRQ = St. George’s Respiratory Questionnaire, Co.VA.Sc = COVID-19 Visual Assessment Scale.

**Table A2:** Univariate and multivariate regression analyses were performed to evaluate the influence of demographics and clinical data during hospitalization on the presence of fibrotic-like changes during follow-up.

	Univariate Analysis			Multivariate Analysis		
Variable	OR	95% CI	*p*-Value	OR	95% CI	*p*-Value
Age	1.007	0.969–1.046	0.732			
Gender (male)	1.303	0.468–3.629	0.613			
BMI	0.949	0.852–1.056	0.337			
Smoking status	1.607	0.556–4.331	0.349			
Vaccination history	0.637	0.196–2.306	0.492			
Length of hospital stay	1.057	1.035–1.088	<0.001	1.062	1.025–1.100	0.01
Co.Va.SC score on admission	2.068	1.149–3.722	0.015	1.385	0.758–2.528	0.289
FiO_2_ ≥ 50%	0.544	0.172–1.722	0.300			
HFNC therapy	0.914	0.345–2.423	0.857			
Intubation	3.1	1.066–9.012	0.038	0.483	0.1–2.338	0.366

BMI = body mass index, FiO_2_ = fraction of inspired oxygen, HFNC = high-flow nasal cannula, Co.VA.Sc = COVID-19 Visual Assessment Scale.

## References

[1] Holshue M.L., DeBolt C., Lindquist S., Lofy K.H., Wiesman J., Bruce H., Spitters C., Ericson K., Wilkerson S., Tural A. (2020). First Case of 2019 Novel Coronavirus in the United States. N. Engl. J. Med..

[2] World Health Organization (WHO) Coronavirus Disease (COVID-19) Pandemic. https://www.who.int/europe/emergencies/situations/covid-19/.

[3] World Health Organization (WHO) Coronavirus Disease (COVID-19) Dashboard. https://covid19.who.int/.

[4] Hoffmann M., Kleine-Weber H., Schroeder S., Krüger N., Herrler T., Erichsen S., Schiergens T.S., Herrler G., Wu N.H., Nitsche A. (2020). SARS-CoV-2 Cell Entry Depends on ACE2 and TMPRSS2 and Is Blocked by a Clinically Proven Protease Inhibitor. Cell.

[5] Wiersinga W.J., Rhodes A., Cheng A.C., Peacock S.J., Prescott H.C. (2020). Pathophysiology, Transmission, Diagnosis, and Treatment of Coronavirus Disease 2019 (COVID-19): A Review. JAMA.

[6] Jin Y., Yang H., Ji W., Wu W., Chen S., Zhang W., Duan G. (2020). Virology, Epidemiology, Pathogenesis, and Control of COVID-19. Viruses.

[7] Gulick R.M., Pau A.K., Daar E., Evans L., Gandhi R.T., Tebas P., Ridzon R., Masur H., Lane H.C., NIH COVID-19 Treatment Guidelines Panel (2024). National Institutes of Health COVID-19 Treatment Guidelines Panel: Perspectives and Lessons Learned. Ann. Intern. Med..

[8] Berlin D.A., Gulick R.M., Martinez F.J. (2020). Severe COVID-19. N. Engl. J. Med..

[9] Yüksel A., Karadoğan D., Hürsoy N., Telatar G., Köse N., Marım F., Kaya I., Er A.B., Erçelik M., Polat D. Methylprednisolone in the treatment of post-COVID-19 Interstitial Lung Disease (STERCOV-ILD). Proceedings of the ERS International Congress.

[10] Myall K.J., Mukherjee B., Castanheira A.M., Lam J.L., Benedetti G., Mak S.M., Preston R., Thillai M., Dewar A., Molyneaux P.L. (2021). Persistent Post-COVID-19 Interstitial Lung Disease. An Observational Study of Corticosteroid Treatment. Ann. Am. Thorac. Soc..

[11] Dhooria S., Chaudhary S., Sehgal I.S., Agarwal R., Arora S., Garg M., Prabhakar N., Puri G.D., Bhalla A., Suri V. (2022). High-dose versus low-dose prednisolone in symptomatic patients with post-COVID-19 diffuse parenchymal lung abnormalities: An open-label, randomised trial (the COLDSTER trial). Eur. Respir. J..

[12] Saiphoklang N., Patanayindee P., Ruchiwit P. (2022). The Effect of Nintedanib in Post-COVID-19 Lung Fibrosis: An Observational Study. Crit. Care Res. Pract..

[13] Bazdyrev E., Rusina P., Panova M., Novikov F., Grishagin I., Nebolsin V. (2021). Lung Fibrosis after COVID-19: Treatment Prospects. Pharmaceuticals.

[14] Cherrez-Ojeda I., Cortés-Telles A., Gochicoa-Rangel L., Camacho-Leon G., Mautong H., Robles-Velasco K., Faytong-Haro M. (2022). Challenges in the Management of Post-COVID-19 Pulmonary Fibrosis for the Latin American Population. J. Pers. Med..

[15] Rajala K., Lehto J.T., Sutinen E., Kautiainen H., Myllärniemi M., Saarto T. (2017). mMRC dyspnoea scale indicates impaired quality of life and increased pain in patients with idiopathic pulmonary fibrosis. ERJ Open Res..

[16] Barr J.T., Schumacher G.E., Freeman S., LeMoine M., Bakst A.W., Jones P.W. (2000). American translation, modification, and validation of the St. George’s Respiratory Questionnaire. Clin. Ther..

[17] Vijayakumar B., Tonkin J., Devaraj A., Philip K.E.J., Orton C.M., Desai S.R., Shah P.L. (2022). CT Lung Abnormalities after COVID-19 at 3 Months and 1 Year after Hospital Discharge. Radiology.

[18] Hansell D.M., Bankier A.A., MacMahon H., McLoud T.C., Müller N.L., Remy J. (2008). Fleischner Society: Glossary of terms for thoracic imaging. Radiology.

[19] Arkoudis N.A., Tsochatzis A., Argentos S., Kontopoulou C., Mademli M., Spiliopoulos S., Oikonomopoulos N. (2021). CT in patients with COVID-19: Imaging patterns, disease extent and evolution; our experience in a Greek reference University Hospital. Hell. J. Radiol..

[20] Arkoudis N.A., Karofylakis E., Moschovaki-Zeiger O., Vrentzos E., Palialexis K., Filippiadis D., Oikonomopoulos N., Kelekis N., Spiliopoulos S. (2022). COVID Visual Assessment Scale (“Co.V.A.Sc.”): Quantification of COVID-19 disease extent on admission chest computed tomography (CT) in the prediction of clinical outcome—A retrospective analysis of 273 patients. Emerg. Radiol..

[21] Karageorgou V., Papaioannou A.I., Kallieri M., Blizou M., Lampadakis S., Sfika M., Krouskos A., Papavasileiou V., Strakosha F., Vandorou K.T. (2023). Patients Hospitalized for COVID-19 in the Periods of Delta and Omicron Variant Dominance in Greece: Determinants of Severity and Mortality. J. Clin. Med..

[22] Wells A.U., Devaraj A., Desai S.R. (2021). Interstitial Lung Disease after COVID-19 Infection: A Catalog of Uncertainties. Radiology.

[23] Han X., Fan Y., Alwalid O., Li N., Jia X., Yuan M., Li Y., Cao Y., Gu J., Wu H. (2021). Six-month Follow-up Chest CT Findings after Severe COVID-19 Pneumonia. Radiology.

[24] McGroder C.F., Zhang D., Choudhury M.A., Salvatore M.M., D’Souza B.M., Hoffman E.A., Wei Y., Baldwin M.R., Garcia C.K. (2021). Pulmonary fibrosis 4 months after COVID-19 is associated with severity of illness and blood leucocyte telomere length. Thorax.

[25] Chen J., Wu J., Hao S., Yang M., Lu X., Chen X., Li L. (2017). Long term outcomes in survivors of epidemic Influenza A (H7N9) virus infection. Sci. Rep..

[26] Matthay M.A., Zemans R.L., Zimmerman G.A., Arabi Y.M., Beitler J.R., Mercat A., Herridge M., Randolph A.G., Calfee C.S. (2019). Acute respiratory distress syndrome. Nat. Rev. Dis. Primers.

[27] Villar J., Zhang H., Slutsky A.S. (2019). Lung Repair and Regeneration in ARDS: Role of PECAM1 and Wnt Signaling. Chest.

